# Cerebrospinal fluid findings of infant tuberculous meningitis: a scoping review

**DOI:** 10.1080/07853890.2022.2123560

**Published:** 2022-09-18

**Authors:** Jie Hou, Xin-Jie Liu, Yu He, Yan-An Zhang, Mao-Shui Wang

**Affiliations:** a Department of Intensive Care, Affiliated Hospital of Shandong University of Traditional Chinese Medicine, Jinan, China; b Department of Pediatrics, Qilu Hospital, Cheeloo College of Medicine, Shandong University, Jinan, China; c Department of Clinical Laboratory, First Affiliated Hospital of Guangxi Medical University, Nanning, China; d Department of Cardiovascular Surgery, Shandong Public Health Clinical Center, Cheeloo College of Medicine, Shandong University, Jinan, China; e Shandong Key Laboratory of Infectious Respiratory Disease, Jinan, China; f Department of Lab Medicine, Shandong Public Health Clinical Center, Cheeloo College of Medicine, Shandong University, Jinan, China

**Keywords:** Infant, tuberculous meningitis, diagnosis, scoping review

## Abstract

**Background:**

Cerebrospinal fluid (CSF) examinations play an important role in the diagnosis of tuberculous meningitis (TBM). However, their yield in the diagnosis of infant TBM remains unclear. This scoping review aims to detail the role of CSF examination for the diagnosis of infant TBM.

**Methods:**

A comprehensive literature search of PubMed, EBSCO, Embase, Scopus, Web of Science, ClinicalTrials.gov, and Cochrane Central Register of Controlled Trials was performed to identify articles published prior to October 14th, 2021. Articles describing the results of CSF exanimations among infant TBM were eligible for inclusion. Data extracted from each study included age, sex, CSF microbiological evidence (such as AFB smear, TB PCR, and TB culture), and routine CSF examinations (such as appearance, red blood cell count, white blood cell count, protein, and glucose).

**Results:**

A total of 98 cases were included in the final analysis. The yield of microbiological methods was listed as follows: CSF AFB smear, 20.5% (9/44); CSF TB culture 47.5% (29/61); CSF TB PCR, 65.0% (26/40); the combination of them, 57.3% (47/82). According to Marais criteria, the positivities of CSF examinations were calculated as follows: WBC count (ref, 50–500/μL), 65.5% (55/84); lymphocyte predominance (ref, >0.5), 75.4% (49/65); total protein (ref, >100 mg/dL), 67.8% (59/87); glucose (ref, <2.2 mmol/L, or CSF/serum ratio < 0.5), 68.2% (58/85).

**Conclusions:**

Our data demonstrated that routine microbiological tools for infant TBM diagnosis have a sensitivity ranging from 20.5% to 65.0%, and most CSF features are non-specific and insufficient to predict a diagnosis of infant TBM. Therefore, further effort is required to develop new tools for infant TBM diagnosis.

**Key messages:**
 Routine microbiological tools (such as acid-fast bacilli smear, PCR, and culture) have an unsatisfactory sensitivity for infant TBM diagnosis, and most CSF features are non-specific and insufficient to predict a diagnosis of infant TBM. Therefore, further effort is required to develop new tools for infant TBM diagnosis.

## Introduction

Tuberculosis (TB) remains a serious public health problem among children. According to the WHO Global Tuberculosis Report (2021), it was estimated that 9.9 million people fell ill with TB in 2020 and children accounted for 11% of them. Moreover, among TB deaths, children were reported to have a significant level [1]. Tuberculous meningitis (TBM) is one of the most serious complications of childhood TB. In a recent report, approximately 10% of children with TBM died, approximately 40% had long-term sequelae, and only half recovered fully [2]. Similarly, in our previous study, approximately 15% of children with TBM had a poor outcome [3]. Fortunately, evidence has accumulated in terms of the management of childhood TB, including TBM [4–7]. Hence, a significant improvement has been observed in recent years [8].

Previously, we summarized the clinicopathological characteristics of infant TB and found that TBM accounted for approximately 20% of TB in infants [9]. Younger age is associated with a poor prognosis of childhood TB [10]. Therefore, although Bacille Calmette-Guérin (BCG) vaccination could prevent TBM, especially in children younger than two years [11,12], infant TBM may pose a more serious challenge. Because infant TBM is rarely reported, its appropriate management remains unclear, and most choices rely on evidence from childhood TBM. The diagnosis of infant TBM remains a serious challenge, as explained by that the following: (1) very little evidence is available for infant TBM in the literature; (2) infant TBM usually has no or few symptoms; and (3) the efficiency of diagnostic tools has not been determined. Hence, for better management of infant TBM, the first step is to evaluate the value of TB diagnostic tools.

CSF examinations play an important role in the diagnosis of TBM. In addition to microbiological methods, other features, such as glucose, protein, and cell count, are routinely evaluated [13]. Until now, their diagnostic usefulness for infant TBM has remained unclear. Therefore, in this study, we conducted a scoping review of the published literature on infant TBM to describe the diagnostic yield of routine CSF examinations, which may improve the current dilemma of infant TBM diagnosis.

## Methods

### Literature search

On October 14th, 2021, we searched several databases, as follows: PubMed, EBSCO, Embase, Scopus, Web of Science, ClinicalTrials.gov, and Cochrane Central Register of Controlled Trials. The full search terms are listed in the Supplementary Materials. Two authors independently screened the reports, and a third arbitrated disagreements between them (HJ, HY, and WMS). The protocol is not registered at PROSPERO because data extraction has been completed.

### Eligibility criteria

Articles describing the results of CSF findings among infant TBM were eligible for inclusion. Infants were defined as ≤24 months. There was no requirement of study design or language. Duplicates were automatically detected by a reference manager and reduced to a single article. The exclusion criteria were unavailable full text, non-TBM, no CSF data, and duplicates. Data extracted from each study included the following items: first author, year of publication, age, sex, Mantoux test or γ-interferon release assay (IGRA), CSF microbiological examinations (such as acid-fast bacilli (AFB) smear, TB PCR, and TB culture), and routine CSF examinations (such as appearance, red blood cell (RBC) count, white blood cell (WBC) count, protein, and glucose).

Confirmed TBM diagnosis is made based on AFB (+) on CSF microscopy, or CSF TB- PCR (+), or *M. tuberculosis* cultured from CSF. A clinical diagnosis of TBM is based on comprehensive analysis of the following items: ① symptoms and signs of meningitis; ② abnormal CSF findings (such as pleocytosis, elevated CSF protein, low CSF glucose, and CSF/serum glucose ratio <0.5); ③ abnormal radiographic features (chest, or cerebral imaging), ④ positive TB assays (such as AFB, PCR, and culture) using non-CSF samples; ⑤ positive responses after anti-TB therapy.

### Statistical analysis

Descriptive methods were used to analyse the data. Continuous variables were reported as median ± interquartile range (IQR) and categorical variables were reported as frequencies (percentages). Continuous variables were also transformed into categorical variables for further analysis if applicable.

## Results

### Baseline characteristics

**Figure 1.**
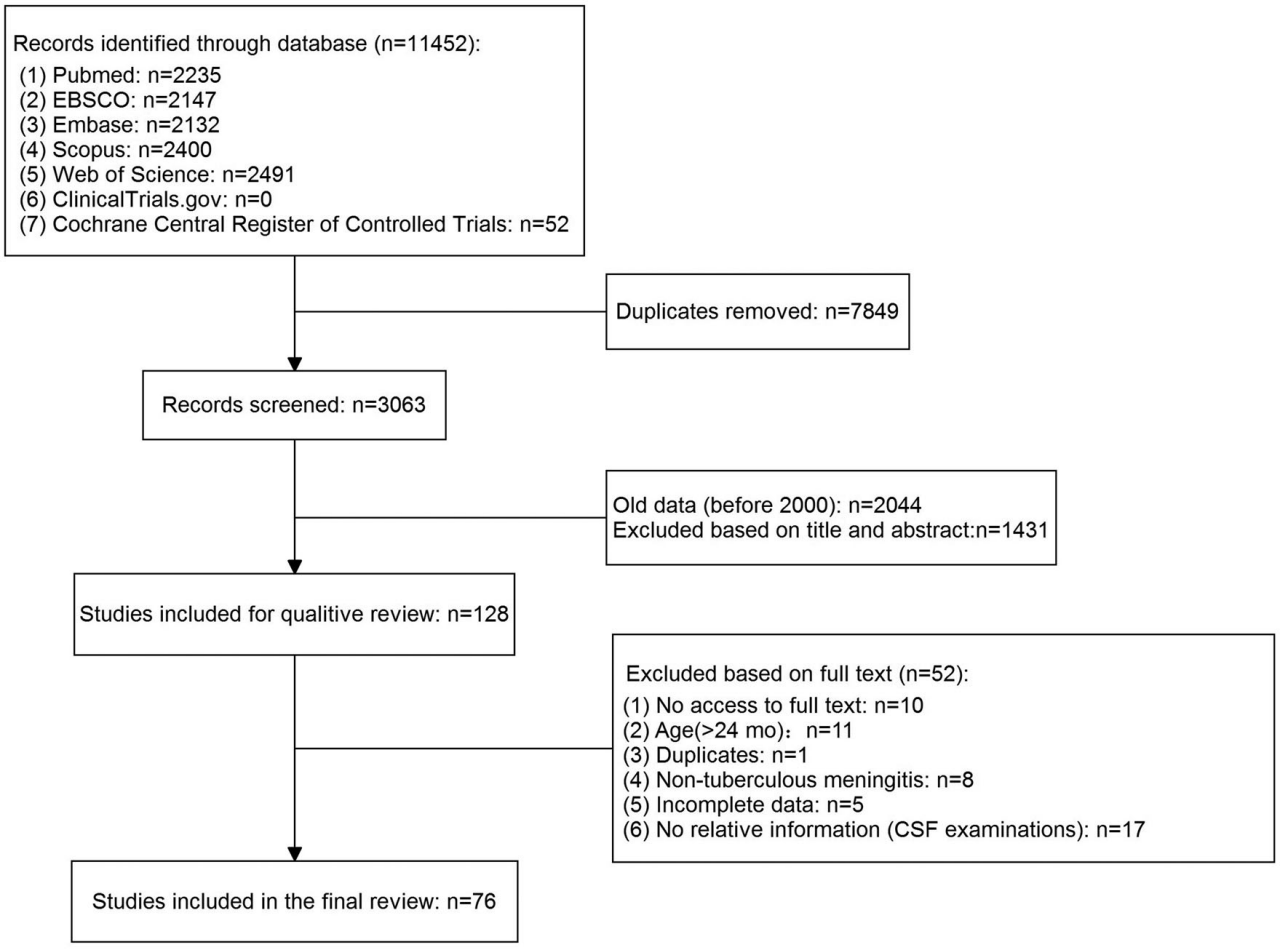
Literature selection.

Seventy-six articles were included in the analysis, and all were case reports. Therefore, risk of bias was not evaluated. A total of 98 cases were included in the final analysis; 47 (48.0%) cases were confirmed as infant TBM, and 51 (52.0%) were clinically diagnosed. The median age was 8.0 (IQR, 5.0, 12.0) months and boys accounted for 53.2% of the infants (50/94). The Mantoux test was positive in 70.3% (45/63) of tested infants, and the IGRA was positive in 72.7% (8/11) of infants. In addition, regarding the TB diagnosis, gastric samples were collected from 53 infants and the corresponding yield of microbiological examinations were as follows: AFB smear (27.6%, 8/29), TB PCR (68.8%, 11/16), TB culture (40%, 20/50), and the combination (AFB, TB-PCR, or TB culture (+); 58.5%, 31/53). The results of other samples are listed in Supplementary Table 1. Figure 1 describes the literature selection process and Table 1 shows the CSF findings of 98 infants with TBM.

**Table 1.** The cerebrospinal fluid findings of infant tuberculous meningitis (TBM, *n* = 98).

	Total (*n* = 98)		Confirmed TBM infants (*n* = 47, 48.0%)		Clinical TBM infants (*n* = 51, 52.0%)	
	Percentages (count, n/n)	Median (IQR)	Percentages (count, n/n)	Median (IQR)	Percentages (count, n/n)	Median (IQR)
Age (months)		8.0 (5.0, 12.0)		11.0 (4.0, 16.0)		8.0 (5.0, 11.0)
Sex, male	53.2% (50/94)		50.0% (23/46)			
TB infection	73.4% (47/64)		64.5% (20/31)		81.8% (27/33)	
Mantoux test (+)	70.3% (45/63)		63.3% (19/30)		78.8% (26/33)	
IGRA (+)	72.7% (8/11)		71.4% (5/7)		75.0% (3/4)	
CSF features						
AFB smear (+)	20.5% (9/44)		39.1% (9/23)		0.0% (0/21)	
PCR (+)	65.0% (26/40)		92.9% (26/28)		0.0% (0/2)	
Culture (+)	47.5% (29/61)		85.3% (29/34)		0.0% (0/5)	
AFB, PCR, or Culture (+)	57.3% (47/82)		100.0% (47/47)		0.0% (0/0)	
Clear appearance	75.0% (9/12)		62.5% (5/8)		0.0% (0/4)	
RBC (/μL)		2 (0, 51)		2.00 (0, 46.5)		2.04 (0, 72.5)
WBC count (50-500/μL)	65.5% (55/84)	101.5 (23.25, 363.25)	73.7% (28/38)	144.0 (43.8, 459.3)	58.7% (27/46)	76.5 (6.75, 209.8)
Lymphocyte predominance (>0.5)	75.4% (49/65)	0.800 (0.545, 0.900)	81.8% (27/33)	0.80 (0.64, 0.92)	68.8% (22/32)	0.74 (0.45, 0.90)
Total Protein (>100 mg/dL)	67.8% (59/87)	133.5 (80.0, 261.0)	75.6% (31/41)	137.0 (100.0, 282.0)	87.5% (28/32)	63.0 (130.0, 219.0)
Glucose	68.2% (58/85)		70.0% (28/40)		66.7% (30/45)	
Glucose (<2.2 mmol/L)	65.9% (56/85)	1.67 (0.94, 2.72)	70.0% (28/40)	1.33 (0.70, 2.42)	62.2% (28/45)	1.11 (1.94, 2.94)
CSF/serum glucose ratio (<0.5)	84.2% (16/19)	0.32 (0.21, 0.40)	90.0% (9/10)	0.31 (0.23, 0.39)	77.8% (7/9)	0.35 (0.18, 0.47)
CSF sodium (mmol/L)		127 (124, 127)		127.00		126.5 (123.5, 128.8)
CSF chloride (mmol/L)		114.5 (112, 119)		114.5 (112.0, 116.8)		117.5 (112.3, 125.0)

AFB: acid-fast bacilli; CSF: cerebrospinal fluid; IQR: interquartile range; PCR: polymerase chain reaction; RBC: red blood cell; WBC: white blood cell.

### Diagnostic yield of CSF examinations

Forty-four infants underwent a CSF AFB smear, and nine (20.5%) of them were positive. CSF TB culture was performed in 61 infants, and the positivity was 47.5% (29/61). The CSF TB PCR appeared to have a relatively high positivity of 65.0% (26/40). Subsequently, the sensitivity of the combination of the three methods was calculated as 57.3% (47/82).

CSF features were described as follows: clear appearance, 75.0% (9/12); RBC count, 2 (0, 51)/μL; WBC count, 101.5 (23.25, 363.25)/μL; lymphocyte proportion, 0.800 (0.545, 0.900); glucose, 1.67 (0.94, 2.72); CSF/serum glucose ratio, 0.32 (0.21, 0.40); sodium, 127 (124, 127) mmol/L; and chloride, 114.5 (112, 119) mmol/L.

Furthermore, according to the Marais criteria [13], the positivities of CSF features were calculated as follows: WBC count (ref, 50–500/μL), 65.5% (55/84); lymphocyte predominance (ref, >0.5), 75.4% (49/65); total protein (ref, >100 mg/dL), 67.8% (59/87); glucose (ref, <2.2 mmol/L, or CSF/serum ratio <0.5), 68.2% (58/85).

## Discussion

To our knowledge, this study is the first study to investigate diagnostic tools for infant TBM diagnosis. In this study, it was demonstrated that the current microbiological tools have a sensitivity ranging from 20.5% to 65.0% for the infant TBM diagnosis, which does not meet the requirements for the rapid diagnosis. In addition, several interesting findings were observed. For example, compared with those in adult TBM, CSF AFB smears in infant TBM have higher positivity. TB PCR appears to have a higher sensitivity for the diagnosis of infant TBM. Most non-specific CSF findings are insensitive.

In infants, the positivity of CSF AFB smears is higher than previously thought. CSF is a kind of paucibacillary sample. In previous reports, the positivity of CSF AFB smears was estimated at 10% [14–16]. However, a higher positivity has been observed. This may be explained by that the following: (1) due to non-specific symptoms and poor awareness of infant TBM, a significant patient delay may occur and result in a high bacterial burden; (2) due to the immature immunity of infants, a high CSF TB burden may be common. Moreover, it is worth noting that a high CSF TB burden means an increased risk of seriously poor outcomes [17]. Two emergency measures should be taken: the first is to increase awareness of infant TBM among the general population; the second is to shorten the diagnostic delay of infant TBM. Therefore, more sensitive methods and sufficient characteristics are required to improve the management of infant TBM.

Compared with other methods, PCR may be a more sensitive method for the confirmation of infant TBM. Compared with that of other populations, the yield of CSF TB PCR in infants appears to have a higher sensitivity (65.0% vs. 40–50%) [18,19]. As mentioned above, patient delay and immature conditions also contribute to the sensitivity difference. However, one limitation should be noted: few cases in the cohort underwent TB PCR examination. This is because the study had a long research period, and PCR assays require an expensive machine, adequate facilities, and skilled operators. In our opinion, further investigation is required to validate this finding. Interestingly, an infant case was recently confirmed to have TBM by Xpert [20]. As a promising TB tool, the role of CSF Xpert in infant TBM should be further evaluated.

According to British guidelines, TBM is suspected if there is CSF leucocytosis (predominantly lymphocytes), the CSF protein is raised, and the CSF/serum glucose is <50% [21]. However, in this study, most CSF features were insensitive for the diagnosis of infant TBM, and all variables had an equal positivity of approximately 70%. Similarly, in a previous report, we found that there is a significant difference in the score for CSF features between children with probable and possible TBM who were confirmed by microbiological methods [22]. This finding confirms that childhood TBM has non-specific symptoms and that more CSF items should be introduced in the revised Marais criteria to improve its diagnostic accuracy for childhood TBM. Regarding infants with a younger age, the insufficient yield of CSF features may be more serious.

Although our study provides valuable insights concerning infant TBM, several limitations must be recognised. First, this study has a retrospective nature and all studies were reported as case reports due to small sample size; thus a selection bias is unavoidable. Second, because the data were collected from previous reports, the issue of missing data should be acknowledged. Third, the sample size was small, and data must be updated if new studies are published. In addition, to save sample size, we decided to perform a scoping review and not systematic review. As known, PICO (population, intervention, comparison, outcome) framework is usually applied to set eligibility criterion of studies for systematic review. In contrast, scoping review has a broader topic nature, and just identify the main concepts of studies and define their PICO elements [23]. Hence, compared with the design of systematic review, the scoping design could include more studies, due to without too much criteria. Finally, a heterogeneous content is another challenge to be faced in the future studies.

## Conclusions

Rapid diagnosis of TBM is one of the most important factors which influencing its outcome. Our data demonstrated that routine microbiological tools for infant TBM diagnosis have a sensitivity ranging from 20.5% to 65.0%, and most non-specific CSF features are insufficient to suspect a diagnosis of infant TBM. Our findings emphasise the importance of new diagnostic tools for infant TBM and more awareness should be raised to decrease patient delay.

## Author contributions

WMS, LXJ, and ZYA designed the study. HJ, WMS, and HY were involved in the literature review and screening, data analysis, interpretation. HJ and WMS wrote the initial paper. All authors read, revised, and approved the final manuscript. In addition, all authors agree to be accountable for all aspects of the work

## Supplementary Material

Supplemental Material

## Data Availability

The data presented in this study are available in supplementary material here.
