# Supplementary material for: Cerebrospinal fluid findings of infant tuberculous meningitis: a scoping review
Source: Ann Med. 2022 Sep 18;54(1):2517–21. doi: 10.1080/07853890.2022.2123560 (PMC9518261; doi:10.1080/07853890.2022.2123560)
Supplement: Supplemental Material [file IANN_A_2123560_SM6582.docx]

**Electronic search strategy**

All search run: October 14, 2021

**1.** **PubMed**

(((“Mycobacterium tuberculosis” OR tuberculosis) and (“Cerebrospinal fluid” OR CSF OR Meningeal)) OR “Tuberculous meningitis” OR TBM OR “Tuberculosis, Meningeal”) and (Infancy OR infant OR infan* OR neonate OR neonat* OR baby OR babies OR fetus OR fetal OR foet* OR toddler OR “new born” OR “new born*” OR newborn OR newborn*)

**2.EBSCO**

| S9 | S7 AND S8 |
| --- | --- |
| S8 | Infancy OR infant OR infan* OR neonate OR neonat* OR baby OR babies OR fetus OR fetal OR foet* OR toddler OR “new born” OR “new born*” OR newborn OR newborn* |
| S7 | S3 OR S4 OR S5 OR S6 |
| S6 | “Tuberculosis, Meningeal” |
| S5 | TBM |
| S4 | “Tuberculous meningitis” |
| S3 | S1 AND S2 |
| S2 | “Cerebrospinal fluid” OR CSF OR Meningeal |
| S1 | “Mycobacterium tuberculosis” OR tuberculosis |

**3.Embase**

| #29 | # 12 AND #28 |
| --- | --- |
| #28 | #13 OR #14 OR #15 OR #16 OR #17 OR #18 OR #19 OR #20 OR #21 OR #22 OR #23 OR #24 OR #25 OR #26 OR #27 |
| #27 | newborn* |
| #26 | newborn |
| #25 | ‘new born*’ |
| #24 | ‘new born’ |
| #23 | toddler |
| #22 | foet* |
| #21 | fetal |
| #20 | fetus |
| #19 | babies |
| #18 | baby |
| #17 | neonat* |
| #16 | neonate |
| #15 | infan* |
| #14 | infant |
| #13 | Infancy |
| #12 | #8 OR #9 OR #10 OR #11 |
| #11 | ‘Tuberculosis, Meningeal’ |
| #10 | TBM |
| #9 | ‘Tuberculous meningitis’ |
| #8 | #3 AND #7 |
| #7 | #4 OR #5 OR #6 |
| #6 | Meningeal |
| #5 | CSF |
| #4 | Cerebrospinal fluid |
| #3 | #1 OR #2 |
| #2 | tuberculosis |
| #1 | “Mycobacterium tuberculosis” |

**4. Scopus**

## ( ( ( TITLE-ABS-KEY ( "Mycobacterium tuberculosis" ) OR TITLE-ABS-KEY ( tuberculosis ) ) AND ( TITLE-ABS-KEY ( "cerebrospinal fluid" ) OR TITLE-ABS-KEY ( csf ) OR TITLE-ABS-KEY ( meningeal ) ) ) OR TITLE-ABS-KEY ( "Tuberculous meningitis" ) OR TITLE-ABS-KEY ( tbm ) OR TITLE-ABS-KEY ( tuberculosis, AND meningeal ) ) AND ( TITLE-ABS-KEY ( infancy ) OR TITLE-ABS-KEY ( infant ) OR TITLE-ABS-KEY ( infan* ) OR TITLE-ABS-KEY ( neonate ) OR TITLE-ABS-KEY ( neonat* ) OR TITLE-ABS-KEY ( baby ) OR TITLE-ABS-KEY ( babies ) OR TITLE-ABS-KEY ( fetus ) OR TITLE-ABS-KEY ( fetal ) OR TITLE-ABS-KEY ( foet* ) OR TITLE-ABS-KEY ( toddler ) OR TITLE-ABS-KEY ( "new born" ) OR TITLE-ABS-KEY ( "new born*" ) OR TITLE-ABS-KEY ( newborn ) OR TITLE-ABS-KEY ( newborn* ) )

**5. Web of Science**

| #7 | #5 AND #6 Databases= WOS, BIOSIS, CSCD, DRCI, DIIDW, KJD, MEDLINE, RSCI, SCIELO Timespan=All years Search language=Auto |
| --- | --- |
| #6 | TOPIC: (Infancy) OR TOPIC: (infant) OR TOPIC: (infan*) OR TOPIC: (neonate) OR TOPIC: (neonat*) OR TOPIC: (baby) OR TOPIC: (babies) OR TOPIC: (fetus) OR TOPIC: (fetal) OR TOPIC: (foet*) OR TOPIC: (toddler) OR TOPIC: (“new born”) OR TOPIC: (“new born*”) OR TOPIC: (newborn) OR TOPIC: (newborn*) Databases= WOS, BIOSIS, CSCD, DRCI, DIIDW, KJD, MEDLINE, RSCI, SCIELO Timespan=All years Search language=Auto |
| #5 | #3 OR #4 Databases= WOS, BIOSIS, CSCD, DRCI, DIIDW, KJD, MEDLINE, RSCI, SCIELO Timespan=All years Search language=Auto |
| #4 | TOPIC: (Tuberculous meningitis) OR TOPIC: (TBM) OR TOPIC: (Tuberculosis, Meningeal) Databases= WOS, BIOSIS, CSCD, DRCI, DIIDW, KJD, MEDLINE, RSCI, SCIELO Timespan=All years Search language=Auto |
| #3 | #1 AND #2 Databases= WOS, BIOSIS, CSCD, DRCI, DIIDW, KJD, MEDLINE, RSCI, SCIELO Timespan=All years Search language=Auto |
| #2 | TOPIC: (Mycobacterium tuberculosis) OR TOPIC: (tuberculosis) Databases= WOS, BIOSIS, CSCD, DRCI, DIIDW, KJD, MEDLINE, RSCI, SCIELO Timespan=All years Search language=Auto |
| #1 | TOPIC: (Cerebrospinal fluid) OR TOPIC: (CSF) OR TOPIC: (Meningeal) Databases= WOS, BIOSIS, CSCD, DRCI, DIIDW, KJD, MEDLINE, RSCI, SCIELO Timespan=All years Search language=Auto |

**6. ClinicalTrials.gov**

(((“Mycobacterium tuberculosis” OR tuberculosis) and (“Cerebrospinal fluid” OR CSF OR Meningeal)) OR “Tuberculous meningitis” OR TBM OR “Tuberculosis, Meningeal”) and (Infancy OR infant OR infan* OR neonate OR neonat* OR baby OR babies OR fetus OR fetal OR foet* OR toddler OR “new born” OR “new born*” OR newborn OR newborn*)

**7. Cochrane Central Register of Controlled Trials (CENTRAL)**

Cochrane Controlled Register of Trials electronic databases

(((“Mycobacterium tuberculosis” OR tuberculosis) and (“Cerebrospinal fluid” OR CSF OR Meningeal)) OR “Tuberculous meningitis” OR TBM OR “Tuberculosis, Meningeal”) and (Infancy OR infant OR infan* OR neonate OR neonat* OR baby OR babies OR fetus OR fetal OR foet* OR toddler OR “new born” OR “new born*” OR newborn OR newborn*)

| Sequence | Authors | Age (months) | Sex | Mantoux test (or IGRA) | Microbiological examinations (CSF) | | | CSF findings | | | | | | | | |
| --- | --- | --- | --- | --- | --- | --- | --- | --- | --- | --- | --- | --- | --- | --- | --- | --- |
|  |  |  |  |  | AFB | PCR | Culture | Appearance | RBC (/ul) | WBC (/ul) | Lymphocyte (%) | Protein (mg/dL) | Glucose (mmol/L) | CSF/serum glucose ratio | Na^+^ (mmol/L) | Cl^-^ (mmol/L) |
| 1 | Janner D, et al. [1] | 8 | M | Positive (15 mm) |  |  |  | - | 89 | 1 | - | 20 | 3.77 | - | - | - |
| 2 | Nomura S, et al. [2] | 18 | F | - | Fite staining (+) | CSF (+); Gastric juice (+) | CSF (-); Tissue (-) | - | - | - | - | - | - | - | - | - |
| 3 | Vasishta R K, et al. [3] | 12 | M | - | - | - | - | - | - | 0 | - | 4000 | 4.4 | 0.8 | - | - |
| 4 | Pejham S, et al. [4] | 2 | F | Positive (12 mm) | CSF (-); Ear discharge (-); Gastric aspirates (-); Cervical lymph node and left mastoid bone biopsy (+) | - | CSF (+); Mastoid tissue (+); Gastric aspirate (+); MiddLe ear fluid (+) | Clear | 0.03 | 0 | 0.48 | 80 | 3.66 | - | - | - |
| 5 | Tung Y R, et al. [5] | 5 | M/unclear | Positive | CSF (-) | - | CSF (-) | - | - | 486 | 0.39 | 990 | 1.39 | - | 124 | - |
| 6 | Tung Y R, et al. [5] | 10 | M/unclear | Positive | CSF (-) | - | CSF (-) | - | - | 100 | 0.2 | 72 | 2.72 | - | 127 | - |
| 7 | Tung Y R, et al. [5] | 8 | M | Positive | CSF (-) | - | CSF (-) | - | - | 175 | 0.07 | 264 | 2.44 | - | 134 | - |
| 8 | Tung Y R, et al. [5] | 5 | M/unclear | Positive | CSF (-) | - | CSF (-) | - | - | 528 | 0.4 | 260 | 0.94 | - | 126 | - |
| 9 | Tung Y R, et al. [5] | 9 | M/unclear | Negative | CSF (-) | - | CSF (+) | - | - | 45 | 0.91 | 282 | 0.83 | - | 127 | - |
| 10 | Tung Y R, et al. [5] | 11 | M/unclear | Positive | CSF (-) | - | CSF (-) | - | - | 35 | 0.47 | 47.5 | 1.39 | - | 122 | - |
| 11 | Tung Y R, et al. [5] | 5 | M/unclear | Positive | CSF (-) | - | CSF (-) | - | - | 185 | 0.45 | 477 | 1.94 | - | 127 | - |
| 12 | Lolekha R, et al. [6] | 20 | F | Positive (15 mm) | Gastric washings (-) | - | CSF (-); Gastric washing (+) | Clear | 0 | 35 | 1 | 932 | 1 | 0.21 | - | - |
| 13 | Crockett M, et al. [7] | 3 | F | - | BALF (+) | BALF (+) | CSF (+); BALF (+) | - | - | - | - | - | - | - | - | - |
| 14 | Geary S, et al. [8] | 24 | F | Negative | CSF (-) | - | CSF (+) | Slightly cloudy | 0 | 0 | 0.82 | 278 | 0.72 | - | - | - |
| 15 | Decarie D, et al. [9] | 2 | M | Positive | - | - | CSF (-); Gastric aspirate (-) | - | - | - | - | - | - | - | - | - |
| 16 | Schoeman J F, et al. [10] | 15 | F | Positive (> 15 mm) | - | - | CSF (-); Gastric aspirate (-) | - | - | - | - | - | - | - | - | - |
| 17 | Spyridis N, Georgouli H, et al. [11] | 4 | F | Positive (16 mm) | Skin lesions (+) | CSF (+) | - | - | - | 70 | 0.97 | 180 | 1.5 | 0.28 | - | - |
| 18 | Chen TT, et al. [12] | 0.3 | M | Negative (+); Negative (-) | CSF (+) | - | - | - | - | 80 | 0.89 | 10600 | 2.63 | - | - | 112 |
| 19 | Goić-Barišić I, et al. [13] | 7 | F | Negative (-); Positive (10mm) | - | - | CSF (+) | - | - | 872 | 0.73 | 300 | 1 | - | - | - |
| 20 | Leaman J. [14] | 11 | M | - | CSF (-) | - | CSF (-); Gastric washing (+) | - | - | 386 | 0.49 | 158 | 0.83 | - | - | - |
| 21 | Meyer S, et al. [15] | 24 | M | - | CSF (+) | CSF (+) | CSF (+) | - | - | 569 | 0.92 | 133 | 1.5 | 0.26 | - | - |
| 22 | Nanda A, et al. [16] | 13 | F | Negative | - | - | CSF (+) | - | - | 68 | 0.64 | 76.8 | 0.7 | - | - | - |
| 23 | Singh M, et al. [17] | 3.5 | M | Positive | - | - | Gastric aspirate (+) | - | - | 40 | Lymphocytic predominance | - | Hypoglycorrachia | - | - | - |
| 24 | Zorn-Olexa C, et al. [18] | 10 | F | Negative (-); Positive (17 mm) | CSF (-) | CSF (-) | Gastric aspirate (+) | - | - | 12 | Predominant lymphocytes | 63 | 38.85 | - | - | 122 |
| 25 | Shah I. [19] | 24 | M | - | - | - | - | - | - | 2800 | 0.9 | 98 | - | - | - | - |
| 26 | Shah I. [19] | 20 | M | Positive | - | - | - | - | - | 110 | 0.9 | 59 | 1.94 | - | - | - |
| 27 | Shah I. [19] | 3 | M | - | Gastric lavage (-) | - | - | - | - | 18 | 0.83 | 146 | 1.22 | - | - | - |
| 28 | Courter J D, et al. [20] | 8 | F | - | - | - | CSF (-); Gastric aspirates (-) | - | - | - | - | - | - | - | - | - |
| 29 | Radmanesh F, et al. [21] | 14 | M | Negative | - | CSF (+) | Tissue (-); Gastric washing (-) | - | - | - | - | 150 | - | - | - | - |
| 30 | Alfayate-Miguélez S, et al. [22] | 8 | M | Positive | - | - | CSF (+) | - | - | 750 | 0.62 | 5077 | 1.11 | - | - | - |
| 31 | Alfayate-Miguélez S, et al. [22] | 11 | F | - | - | - | CSF (+) | - | - | 420 | 0.88 | 4800 | 1 | - | - | - |
| 32 | Basu S, et al. [23] | 4 | M | Negative | CSF (-) | CSF (+) | CSF (-) | - | - | 180 | 0.9 | 300 | 1.3 | 0.39 | - | - |
| 33 | Singh D K, et al. [24] | 12 | F | - | - | CSF (+) | - | Colorless | - | - | Marked lymphocytic pleocytosis | 31 | 3.55 | 0.74 | - | - |
| 34 | Wen L S, et al. [25] | 16 | F | - | - | - | CSF (+); Gastric aspirate (+) | - | - | - | - | - | - | - | - | - |
| 35 | Zaki S, et al. [26] | 7 | F | Positive | - | - | - | - | - | 0 | 0.6 | 160 | 2.11 | - | - | - |
| 36 | Gupta K, et al. [27] | 5 | M | - | Gastric lavage (+) | CSF (-); Lung and brain tissues (+) | CSF (-) | - | - | 0.02 | - | 200 | 0.83 | - | - | - |
| 37 | van Toorn R, et al. [28] | 21 | F | Positive (>20 mm) | - | - | - | Clear and colourless | - | 382 | - | 95 | 4.1 | - | - | - |
| 38 | Zaki S A, et al. [29] | 6 | F | Positive | - | - | - | - | - | 0 | 0.4 | 160 | 2.11 | - | - | - |
| 39 | Katragkou A, et al. [30] | 18 | M | Negative; IGRA (indetermine) | - | CSF (+) | CSF (+) | - | - | 100 | 0.8 | 82 | 0.94 | 0.13 | - | - |
| 40 | Martínez-Antón A, et al. [31] | 16 | F | - | CSF (-); Respiratory sample (-) | CSF (+); Respiratory sample (+) | CSF (-) | - | - | 0 | - | 20 | 4.61 | - | - | - |
| 41 | Smith B B, et al. [32] | 17 | M | Positive (5 mm); QFT(+) | - | CSF (+); Endotracheal aspirate specimens (+) | CSF (+) | - | 42 | 31 | 0.41 | 100 | 14.99 | - | - | - |
| 42 | Smith B B, et al. [32] | 18 | M | Positive (12 mm) | - | CSF (+); BALF (+); Gastric aspirate (+) | CSF (+); Gastric aspirate (+); BALF (+) | Turbid | 0 | 211 | 0.38 | 137 | 5.55 | - | - | - |
| 43 | Yilmaz R, et al. [33] | 7 | F | Negative (1 mm) | CSF (-) | - | - | - | 67 | 69 | - | 48.5 | 2.33 | 0.37 | - | - |
| 44 | Birnbaum G D, et al. [34] | 22 | F | - | - | - | CSF (+); Gastric aspirate (+) | - | - | - | - | - | - | - | - | - |
| 45 | Kumar S, et al. [35] | 3 | M | Negative | CSF (-); Gastric lavages (-); Brain biopsy (+); Post-mortem examination: liver (+), spleen (+), kidneys (+), and lungs (+) | - | CSF (+); Gastric ﬂuid (-) | - | - | 400 | 0.8 | 119 | 1.33 | 0.29 | - | - |
| 46 | Palm S, et al. [36] | 12 | F | Positive | - | - | CSF (+); Gastric ﬂuid (+) | - | 60 | 397 | - | 221 | 2.39 | - | - | - |
| 47 | Dayal D, et al. [37] | 10 | F | Positive (10 mm) | CSF (-); Gastric aspirate (-) | CSF (-) | CSF (-) | - | - | 160 | 0.8 | 98 | 0.83 | - | - | - |
| 48 | Kumar M R, et al. [38] | 12 | F | - | - | - | - | - | - | 0 | 0.6 | 540 | 1.67 | 0.35 | - | - |
| 49 | Hoyos-Orrego Á, et al. [39] | 1 | M | Negative | CSF (-); Gastric aspirate (+) | CSF (+) | CSF (-); Gastric aspirate (+) | - | - | - | - | - | - | - | - | - |
| 50 | Xu Y, et al. [40] | 12 | M | Positive; T-SPOT.TB (-) | - | - | CSF (-) | - | - | 30 | 0.6 | 117 | 1 | - | - | 113 |
| 51 | Cifuentes Y, et al. [41] | 1 | M | - | Gastric lavage (-) | CSF (+); Urine (+); Serum (-) | - | - | - | 13 | 0.98 | 77.7 | - | - | - | - |
| 52 | Zak H K, et al. [42] | 0.33 | M | - | CSF (+); Gastric aspirate (-) | - | - | - | Many RBC | - | - | 300 | 1.5 | 0.36 | - | - |
| 53 | Kratimenos P, et al. [43] | 6 | F | - | - | - | - | Colorless | 8 | 84 | - | 154 | 3.33 | 0.43 | - | - |
| 54 | Shah I, et al. [44] | 18 | M | Positive | - | CSF (+) | CSF (+) | - | - | 193 | 1 | 90 | 1.39 | - | - | - |
| 55 | Singh S N, et al. [45] | 8 | M | Positive (15 mm) | - | - | - | - | - | 120 | - | 200 | 1.11 | - | - | - |
| 56 | Patil S, et al. [46] | 24 | M | Positive (14 mm) | Gastric aspirate (+); Sputum (+) | - | - | - | - | 428 | 0.9 | 110 | 3.11 | 0.51 | - | - |
| 57 | Paul M, et al. [47] | 8 | F | Positive (10 mm) | CSF (-); Gastric lavage (-) | CSF (-) | CSF (+); Gastric lavage (+) | - | 2 | 0.07 | 0.92 | 117 | 0.5 | 0.12 | - | - |
| 58 | Said M, et al. [48] | 11 | M | Positive; QFT (+) | Endotracheal aspirate sample (+) | - | CSF (+) | - | - | 134 | 0.66 | 742 | 0.03 | - | - | - |
| 59 | Anandakrishnan P, et al. [49] | 8 | F | Negative | CSF (-); Lymph nodes biopsy: (+) | Lymph nodes biopsy: Xpert (+) | Lymph nodes (+) | - | - | 0 | - | 67000 | 3.2 | - | - | - |
| 60 | Dhawan S R, et al. [50] | 10 | - | Positive | - | - | CSF (-); Gastric aspirate (-) | - | - | 110 | 0.8 | 130 | 1.11 | 0.23 | - | - |
| 61 | Dhawan S R, et al. [50] | 3 | - | Positive | - | - | CSF (-); Gastric aspirate (-) | - | - | 275 | 0.93 | 411 | 0.83 | 0.14 | - | - |
| 62 | Dhawan S R, et al. [50] | 4 | - | Negative | - | - | CSF (-); Gastric aspirate (-) | - | - | 60 | Predominant lymphocytes | 134 | 2.05 | - | - | - |
| 63 | Nguyen P T K, et al. [51] | 5 | M | - | Gastric aspirate (-) | CSF: Xpert (+); Gastric aspirate: Xpert (-) | - | Clear | - | 207 | 0.25 | 830 | 0.7 | - | - | - |
| 64 | Ramírez M, et al. [52] | 11 | M | Negative | CSF (-); Gastric aspirate (-); Brain tissues (+) | CSF (-) | CSF (-); Gastric aspirate (-) | - | 0.08 | 0 | - | 22.5 | 5.05 | - | - | - |
| 65 | Rosales Magallanes G. [53] | 11 | M | Negative (-); QFT(+) | CSF (-) | CSF (-) | - | Clear | 0 | 0 | 0 | 219 | 0.67 | - | - | - |
| 66 | Huynh J, et al. [54] | 23 | F | Positive (20mm); IGRA (+) | - | CSF (+); Gastric aspirates: Xpert Ultra (+) | - | Clear | 2 | 1280 | 0.97 | 2.3 | 1.7 | - | - | - |
| 67 | Andriescu E C, et al. [55] | 10 | F | Positive (15 mm); T-SPOT.TB (+) | CSF (-) | - | CSF (-) | - | - | 218 | 0.89 | 39 | 2.78 | - | - | - |
| 68 | Bilasco A, et al. [56] | 21 | F | - | - | CSF (-) | CSF (-) | - | - | Lymphocytosis | - | - | Decreased glucose | - | - | - |
| 69 | Foreman E, et al. [57] | 8 | F | Negative (-); Positive (+) | Sputum (+) | - | - | - | 128 | 14 | - | 50 | 1.89 | - | - | - |
| 70 | Locham J, et al. [58] | 1 | F | - | Gastric aspirate (-); Liver biopsy (+) | - | Gastric aspirate (-) | - | - | Leucocytosis | Predominant lymphocytes, | Raised proteins | - | - | - | - |
| 71 | Morais C G, et al. [59] | 18 | F | - | - | CSF (+); Bronchoalveolar lavage (+); Gastric aspirate (+) | CSF (+) | - | Pleocytosis | - | Polymorphonuclear neutrophils predominance | - | Hypoglycorrhachia. | - | - | - |
| 72 | Sánchez-Códez M I, et al. [60] | 10 | F | Negative (0 mm); IGRA(+) | - | CSF (+); Gastric aspirates: Xpert (+) | - | - | - | 25 | - | Normal | Normal | - | - | - |
| 73 | Cyr G S, et al. [61] | 3 | F | - | CSF (-) | - | Gastric aspirate (+) | - | 4 | 5 | - | 36 | 2.94 | - | - | - |
| 74 | Cyr G S, et al. [61] | 8 | M | - | CSF (-) | - | Gastric aspirate (+) | - | 0 | 1 | - | 23 | 2.94 | - | - | - |
| 75 | Dreesman A, et al. [62] | 2 | M | - | - | BALF (+) | CSF (-); Gastric aspirate (+); BALF (+) | - | - | - | - | Normal. | Normal | - | - | - |
| 76 | Furuichi M, et al. [63] | 16 | M | IGRA (-) | - | CSF (-); CSF (+); ventricular catheters and brain tissues (+) | CSF (-) | - | - | 57 | 0.88 | 254 | 1.72 | 0.4 | - | 115 |
| 77 | Gigi R M S, et al. [64] | 4 | F | - | - | - | Gastric ﬂuid (+) | - | - | 110 | 0.87 | 105 | 1.4 | - | - | - |
| 78 | Kasinathan A, et al. [65] | 11 | F | Positive (18mm) | - | CSF: Xpert (-); Gastric aspirate: Xpert (+) | - | - | - | 270 | 0.9 | 173 | 1.67 | - | - | - |
| 79 | Lingesan K, et al. [66] | 10 | F | - | CSF (-); Gastric aspirate (-) | CSF (-); Gastric aspirate: Xpert (-) | - | - | - | 30 | - | 58 | 1.72 | - | - | - |
| 80 | Sun J, et al. [67] | 0.4 | F | - | CSF (+); Gastric aspirate (+); Sputum (+) | CSF (+) | Gastric aspirate (+); Sputum (+) | - | - | 71 | - | 113 | 2 | 0.32 | - | - |
| 81 | Yang J, et al. [68] | 7 | F | - | CSF (-); Mediastinal tissues (+) | CSF (-); Mediastinal mass (+) | CSF (-) | - | - | - | Monocytic predominant pleocytosis | Elevated protein | - | - | - | - |
| 82 | Du J, et al. [69] | 0.83 | - | Positive (+); T-SPOT.TB (+) | CSF (+); Gastric ﬂuid (+) | CSF: Xpert(+); Gastric ﬂuid: Xpert (+) | Gastric ﬂuid (+) | - | - | 326000 | 0.72 | Pro↑ | Glu ↓ | - | - | Cl↓ |
| 83 | Ngoc D V, et al. [70] | 3 | M | - | - | CSF (+); Sputum (+) | - | Yellow | - | 40 | - | 300 | 0.56 | - | - | - |
| 84 | Khambati N, et al. [71] | 5 | M | - | - | CSF: Xpert (-); BALF: Xpert (+) | CSF (-) | - | 0 | 8000 | - | 26440 | 0.02 | 0.07 | - | - |
| 85 | Yang J H, Vuong K T, MoodLey A, et al. [72] | 7 | F | Positive (10 mm); QFT (+) | CSF(-); Mediastinal mass (+) | CSF (-) | CSF (-); Mediastinal mass (+) | - | - | 101 | 0.74 | 120 | 3.16 | - | - | - |
| 86 | Pereira R M, Tresoldi A T, Hessel G. [73] | 5 | M | - | Gastric ﬂuid (+) | - | Gastric ﬂuid (+) | - | - | 102 | 0.56 | 80 | 2.16 | - | - | - |
| 87 | Moraes M, González S, García A, et al. [74] | 5 | M | - | CSF (+) | - | - | Clear | - | 0 | - | 115 | 0.01 | - | - | - |
| 88 | Kondo S, Miyagawa T. [75] | 12 | M | Positive (13mm) | CSF (-); Gastric ﬂuid (-) | - | CSF (+); Gastric ﬂuid (+) | - | - | 822 | - | 242 | 0.61 | - | - | - |
| 89 | Kondo S, et al. [76] | 4 | F | Negative (0 mm) | CSF (-); Gastric ﬂuid (-) | - | CSF (+); Gastric ﬂuid (-) | - | - | 126 | 0.6 | 119 | 2.39 | - | - | 119 |
| 90 | Kondo S, et al. [76] | 4 | M | Negative (0 mm) | CSF (-); Gastric ﬂuid (-) | CSF (-); Gastric ﬂuid (-) | CSF (-); Gastric ﬂuid (-) | - | - | 56 | 0.95 | 63 | 2.66 | - | - | 126 |
| 91 | Kondo S, et al. [76] | 6 | M | Positive (10 mm) | CSF (+); Gastric ﬂuid (+) | CSF (+); Gastric ﬂuid (+) | CSF (+); Gastric ﬂuid (-) | - | - | 103 | 0.67 | 67 | 2.72 | - | - | 113 |
| 92 | Kondo S, et al. [76] | 7 | M | Negative (0 mm) | CSF (+); Gastric ﬂuid (+) | CSF (+); Gastric ﬂuid (+) | CSF (+); Gastric ﬂuid (-) | - | - | 154 | 0.98 | 118 | 0.94 | - | - | 115 |
| 93 | Kondo S, et al. [76] | 8 | M | Negative (0 mm) | CSF (-); Gastric ﬂuid (-) | - | CSF (-); Gastric ﬂuid (-) | - | - | 357 | 0.97 | 171 | 1.33 | - | - | 112 |
| 94 | Kondo S, et al. [76] | 10 | F | - | CSF (-); Gastric ﬂuid (-) | CSF (+); Gastric ﬂuid (+) | CSF (+); Gastric ﬂuid (-) | - | - | 443 | 0.95 | 165 | 3.72 | - | - | 119 |
| 95 | Kondo S, et al. [76] | 11 | F | Positive (9 mm) | CSF (-); Gastric ﬂuid (-) | - | CSF (+); Gastric ﬂuid (-) | - | - | 508 | 0.8 | 131 | 0.44 | - | - | 112 |
| 96 | Kondo S, et al. [76] | 11 | M | Positive (5 mm) | CSF (-); Gastric ﬂuid (-) | CSF (+); Gastric ﬂuid (-) | CSF (+); Gastric ﬂuid (-) | - | - | 597 | 0.75 | 230 | 0.67 | - | - | 109 |
| 97 | Kondo S, et al. [76] | 11 | M | Positive (13 mm) | CSF (-); Gastric ﬂuid (-) | CSF (-); Gastric ﬂuid (-) | CSF (+); Gastric ﬂuid (-) | - | - | 823 | 0.5 | 242 | 0.61 | - | - | 114 |
| 98 | Kondo S, et al. [76] | 16 | F | Positive (11 mm) | CSF (+); Gastric ﬂuid (+) | CSF (+); Gastric ﬂuid (+) | CSF (+); Gastric ﬂuid (-) | - | - | 183 | 0.67 | 116 | 2.44 | - | - | 116 |

**References**

1. Janner D, Kirk S, McLeary M. Cerebral tuberculosis without neurologic signs and with normal cerebrospinal fluid. Pediatr Infect Dis J. 2000;19:763-764.

2. Nomura S, Akimura T, Kitahara T, Nogami K, Suzuki M. Surgery for expansion of spinal tuberculoma during antituberculous chemotherapy: A case report. Pediatric Neurosurgery. 2001;35:153-157.

3. Vasishta RK, Kakkar N, Singhi P, Banjerjee AK. Pathological case of the month. Archives of Pediatrics & Adolescent Medicine. 2001;155:517-518.

4. Pejham S, Altman R, Li KI, Munoz JL. Congenital tuberculosis with facial nerve palsy. Pediatr Infect Dis J. 2002;21:1085-1086.

5. Tung Y-R, Lai M-C, Lui C-C, Tsai K-L, Huang L-T, Chang Y-C, Huang S-C, Yang SN, Hung P-L. Tuberculous meningitis in infancy. Pediatric Neurology. 2002;27:262-266.

6. Lolekha R, Chokephaibulkit K, Vanprapar N, Phongsamart W, Chearskul S. Breakthrough neurological manifestation during appropriate antituberculous therapy of miliary tuberculosis. Southeast Asian J Trop Med Public Health. 2003;34:634-635.

7. Crockett M, King SM, Kitai I, Jamieson F, Richardson S, Malloy P, Yaffe B, Reynolds D, Hellmann J, Cutz E, Matlow A. Nosocomial transmission of congenital tuberculosis in a neonatal intensive care unit. Clinical Infectious Diseases. 2004;39:1719-1723.

8. Geary S, Agnew M. 2-year-old with tuberculosis meningitis: a case study. Journal of Neuroscience Nursing. 2004;36:90-94.

9. Décarie D, Grenier JL, Allard A. Outbreak of tuberculosis in the Laurentian region, 2005. Can Commun Dis Rep. 2006;32:226-229.

10. Schoeman JF, Fieggen G, Seller N, Mendelson M, Hartzenberg B. Intractable intracranial tuberculous infection responsive to thalidomide: report of four cases. J Child Neurol. 2006;21:301-308.

11. Spyridis N, Georgouli H, Tsoukatou T, Sakou I, Tsolia M, Miriokefalitakis N, Spyridis P. Severe disseminated tuberculosis in a 4-month-old infant initially presenting with multiform cutaneous lesions. Scand J Infect Dis. 2006;38:306-308.

12. Chen T-t, Duan H-y, Chen J. [Tuberculous meningitis in a newborn infant]. Zhonghua er ke za zhi = Chinese journal of pediatrics. 2007;45:472-473.

13. Goić-Barišić I, Pavlov N, Ivić I, Tonkić M, Barišić I, Dragišić-Ivulić S, Punda-Polić V. Pulmonary tuberculosis with meningitis in a 7-month-old infant. Journal of Pediatric Infectious Diseases. 2007;2:51-54.

14. Leaman J. Meningitis in an infant: all that's aseptic is not viral. Jaapa. 2007;20:26, 29-31.

15. Meyer S, Shamdeen MG, Wüllenweber J, Hermann M, Gottschling S, Struffert T, Gortner L, Klotz M. Simultaneous tuberculous meningoencephalitis in two siblings. Wien Med Wochenschr. 2007;157:37-42.

16. Nanda A, Nanda M, Dvorak R, Al-sabah H, Alsaleh QA. Bullous pemphigoid (BP) in an infant complicated by tuberculous meningoencephalitis. International Journal of Dermatology. 2007;46:964-966.

17. Singh M, Kothur K, Dayal D, Kusuma S. Perinatal tuberculosis a case series. Journal of Tropical Pediatrics. 2007;53:135-138.

18. Zorn-Olexa C, Laugel V, Martin AdS, Donato L, Fischbach M. Multiple intracranial tuberculomas associated with partial status epilepticus and refractory infantile spasms. Journal of Child Neurology. 2008;23:459-462.

19. Shah I. Steroid therapy in children with tuberculous meningitis. Scand J Infect Dis. 2009;41:532-534.

20. Courter JD, Girotto JE, Lobato MN, Orcutt D, Burke M, Feder Jr HM, Krause PJ, Cohen-Abbo A, Salazar JC. Intravenous streptomycin for treatment of Mycobacterium tuberculosis meningitis in an infant. Pharmacotherapy. 2010;30:481e-484e.

21. Radmanesh F, Nejat F, El Khashab M. Cerebral infarction as the first presentation of tuberculosis in an infant: a case report. J Microbiol Immunol Infect. 2010;43:249-252.

22. Alfayate-Miguélez S, Martínez-Lage-Azorín L, Marín-Vive L, García-Martínez S, Almagro MJ, Martínez-Lage JF. Normal ventricular-CSF may confound the diagnosis of tuberculous meningitis hydrocephalus. Neurocirugia. 2011;22:157-161.

23. Basu S, Kumar A, Das BK. Perinatal tuberculosis: two unusual cases. Ann Trop Paediatr. 2011;31:81-86.

24. Singh DK, Singh N. Paroxysmal autonomic instability with dystonia in a child: rare manifestation of an interpeduncular tuberculoma. Pediatr Neurosurg. 2011;47:275-278.

25. Wen LS, Noble JA. A case of a young girl with fever and seizure. Acad Emerg Med. 2011;18:e86-92.

26. Zaki SA, Lad V, Shanbag P. Diagnostic dilemma in a child with tuberculous meningitis on haloperidol therapy. Toxicology International. 2011;18:173-174.

27. Gupta K, Radotra BD, Suri D, Sharma K, Saxena AK, Singhi P. Mycotic aneurysm and subarachnoid hemorrhage following tubercular meningitis in an infant with congenital tuberculosis and cytomegalovirus disease. J Child Neurol. 2012;27:1320-1325.

28. van Toorn R, Rabie H, Dramowski A, Schoeman JF. Neurological manifestations of TB-IRIS: a report of 4 children. Eur J Paediatr Neurol. 2012;16:676-682.

29. Zaki SA, Lad V, Shanbag P. Cerebral salt wasting following tuberculous meningoencephalitis in an infant. Annals of Indian Academy of Neurology. 2012;15:148-150.

30. Katragkou A, Antachopoulos C, Hatziagorou E, Sdougka M, Roilides E, Tsanakas J. Drug-resistant tuberculosis in two children in Greece: Report of the first extensively drug-resistant case. European Journal of Pediatrics. 2013;172:563-567.

31. Martínez-Antón A, Montoro S, Gavela T, Ruiz-Juretschke F. [Tuberculous meningitis hydrocephalus with normal ventricular cerebrospinal fluid]. An Pediatr (Barc). 2013;79:128-130.

32. Smith BB, Hazelton BJ, Heywood AE, Snelling TL, Peacock KM, Macartney KK. Disseminated tuberculosis and tuberculous meningitis in Australian-born children; case reports and review of current epidemiology and management. J Paediatr Child Health. 2013;49:E246-250.

33. Yilmaz R, Kundak AA, Sezer T, Özer S, Esmeray H, Kazanci NO. Idiopathic infantile hypercalcemia or an extrapulmonary complication of tuberculosis? Tuberkuloz ve Toraks. 2013;61:43-46.

34. Birnbaum GD, Marquez L, Hwang KM, Cruz AT. Neurologic deterioration in a child undergoing treatment for tuberculosis meningitis. Pediatr Emerg Care. 2014;30:566-567.

35. Kumar S, Kumar R, Radotra BD, Singh M. Tubercular ventriculitis: an uncommon entity. Indian J Pediatr. 2014;81:608-610.

36. Palm S, Balan A, Ramji F. Persistent fever in an infant with meningitis. Journal of Investigative Medicine. 2014;62:471.

37. Dayal D, Didel SR, Agarwal S, Sachdeva N, Singh M. Acute Hypercalcaemia and Hypervitaminosis D in an Infant with Extra Pulmonary Tuberculosis. J Clin Diagn Res. 2015;9:Sd03-04.

38. Kumar RM, Saini L, Kaushik JS, Chakrabarty B, Kumar A, Gulati S. A Combination of Moyamoya Pattern and Cerebral Venous Sinus Thrombosis: A Case of Tubercular Vasculopathy. J Trop Pediatr. 2015;61:393-396.

39. Hoyos-Orrego Á, Trujillo-Honeysberg M, Diazgranados-Cuenca L. Congenital Tuberculosis as a Result of Disseminated Maternal Disease: Case Report. Tuberc Respir Dis (Seoul). 2015;78:450-454.

40. Xu Y, Wan L, Ning J, Guo W, Ren L. Paroxysmal Sympathetic Hyperactivity in a Child with Tuberculous Meningitis A Case Study and Review of Related Literature. West Indian Medical Journal. 2015;64:543-547.

41. Cifuentes Y, Murcia MI, Piar J, Pardo P. Cerebral microcalcifications in a newborn with congenital tuberculosis. Biomedica. 2016;36:22-28.

42. Khorsand Zak H, Mafinezhad S, Haghbin A. Congenital Tuberculosis: A Newborn Case Report With Rare Manifestation. Iran Red Crescent Med J. 2016;18:e23572.

43. Kratimenos P, Koutroulis I, Fruscione M, Adigun H, DeGroote R, Fisher MC. Infantile Tubercular Meningitis With Brain Infarct. Pediatr Emerg Care. 2016;32:95-97.

44. Shah I, Goyal A. Childhood multi-drug resistant tuberculosis with paradoxical granulomas – role of steroids. Infectious Diseases. 2016;48:862-863.

45. Singh SN, Bhatt TC, Kumar S, Chauhan V, Pandey A. A Case of Cervical Spine Tuberculosis in an Infant. J Clin Diagn Res. 2016;10:Td03-05.

46. Patil S, Nagargoje N, Choudhary CR. Tuberculous Meningitis in Two-Year-Old Male Boy: Evidence of Recent Transmission from Mother to Baby. Anneden Çocuğa Bulaşmanın Kanıtı Olarak Tüberküloz Menenjitli İki Yaşında Erkek Çocuk. 2017;6:5-11.

47. Paul M, Shehab K, Nguyen T. Case 1: Progressive Hypotonia and Decreased Alertness in an 8-month-old Girl. Pediatr Rev. 2017;38:383.

48. Said M, Uppal P, Bye A, Palasanthiran P. Unusual case of tuberculous meningitis with discordant ventricular and lumbar cerebrospinal fluid; lessons in the era of world-wide migration. J Paediatr Child Health. 2018;54:93-95.

49. Anandakrishnan P, Khoo TB. Unusual case of cerebral demyelination and bilateral optic neuritis in an infant with suppurative BCG lymphadenitis. BMJ Case Rep. 2018;2018.

50. Dhawan SR, Sahu JK, Sankhyan N, Vyas S, Singhi PD. Infantile Tuberculous Meningitis Complicated by West Syndrome. J Pediatr Neurosci. 2018;13:237-240.

51. Nguyen PTK, Thai TTB, Huynh J, Marais BJ. An Infant with Xpert(®) Confirmed TB Meningitis in Central Viet Nam. J Clin Med. 2018;7.

52. Ramírez M, Cortés E, Betancur J, Garcés C. Cerebral tuberculosis without meningitis in a immunocompetent child. Rev Chilena Infectol. 2018;35:207-212.

53. Francisco Rosales-Magallanes G. Meningitis tuberculosa en un lactante, a propósito de la aplicación de BCG a migrantes de zonas endémicas. Revista Mexicana de Pediatria. 2018;85:222-225.

54. Huynh J, Vosu J, Marais BJ, Britton PN. Multidrug-resistant tuberculous meningitis in a returned traveller. J Paediatr Child Health. 2019;55:981-984.

55. Andriescu EC, Khetan NG, Mazur L, Smith KC. Tuberculosis Meningitis in a 10-Month-Old Living in an Immigrant Township. Clin Pediatr (Phila). 2019;58:1341-1344.

56. Bilasco A, Cirt R, Florea S, Draganescu A, Vasile M, Kouris C, Negulescu C, Luminos M. Pulmonary miliary tuberculosis in a toddler after initial presentation with tuberculous meningitis. Archives of Disease in Childhood. 2019;104:A58.

57. Foreman E, Raju SS. COMMUNICATING HYDROCEPHALUS IN AN INFANT WITH TUBERCULOSIS MENINGITIS. Journal of Investigative Medicine. 2019;67:483-483.

58. Locham J, Chandra Shaw S, Dalal SS, Gupta R. Congenital tuberculosis in a neonate. Medical Journal Armed Forces India. 2019;75:96-98.

59. Morais CG, Pereira-Nunes J, Reis-Melo A, Tavares M, Faria C, Oliveira J, Miguel N, Pinto-Carvalho I. Cerebral vasculitis in meningeal tuberculosis: A case study. European Journal of Pediatrics. 2019;178:1710.

60. Sánchez-Códez MI, Lubián-Gutiérrez M, Fernández-Bravo C, Ley-Martos M. Pediatric miliary tuberculosis presenting with stroke: contribution to the paper "Tuberculosis of the central nervous system in children". 2019. p. 1273-1275.

61. St Cyr G, Starke JR. Multiple Cranial Tuberculomas Without Meningitis in Two Infants With Miliary Tuberculosis. Pediatr Infect Dis J. 2019;38:e337-e339.

62. Dreesman A, Stévart O, Adler C, Mathys V, Mouchet F. Recurrent tuberculosis in a young child. Pediatric Infectious Disease Journal. 2020;39:E207-E209.

63. Furuichi M, Mori F, Uejima Y, Sato S, Kurihara J, Kawano Y, Suganuma E. A case of Mycobacterium bovis Bacillus Calmette-Guérin (BCG) strain meningitis and ventriculitis following BCG vaccination. Int J Infect Dis. 2020;100:373-376.

64. Gigi RMS, Rieder HL, Padayatchi N. When to start treatment? Dilemma illustrated by a paediatric case of extensively drug-resistant tuberculosis of the central nervous system. S Afr Med J. 2020;110:846-849.

65. Kasinathan A, Serane VK, Palanisamy S. Tuberculous meningitis manifesting with neuroregression in a eleven month child. Indian J Tuberc. 2020;67:136-138.

66. Lingesan K, Maya M, Ramamoorthy JG, Karunakar P, Gunasekaran D, Thangavel NP, Kumar RS, Kumar VR. Tuberculous Meningitis Fitting the Shoes of Incomplete Kawasaki Disease. Indian Journal of Pediatrics. 2020;87:1086-1087.

67. Sun J-m, Wang C, Jin D-q, Deng F. Fatal congenital tuberculosis owing to late diagnosis of maternal tuberculosis: case report and review of congenital tuberculosis in China. Paediatrics & International Child Health. 2020;40:194-198.

68. Yang J, Vuong K, Moodley A, Chen D. An Unusual Case of TB-associated Acute Demyelinating Encephalomyelitis in a 7-month-old Infant. Neurology. 2020;94.

69. Du J, Dong S, Jia S, Zhang Q, Hei M. Clinical characteristics and post-discharge follow-up analyses of 10 infants with congenital tuberculosis: A retrospective observational study. Pediatric Investigation. 2021;5:86-93.

70. Duc LA, Ngoc DV, Trung NN, Sang NV, Ninh TP, Giang TV, Tra My TT, Hoa T, Duc NM. Miliary brain tuberculosis in an infant. Radiol Case Rep. 2021;16:2882-2885.

71. Khambati N, Hou M, Kelly D, Song R. Fatal tuberculous meningitis in an infant presenting with seizures in the UK. BMJ Case Rep. 2021;14.

72. Yang JH, Vuong KT, Moodley A, Chuang NA, Chen DY. A Case of Tuberculosis-Associated Acute Disseminated Encephalomyelitis in a Seven-Month-Old Infant. Cureus. 2021;13:e16299.

73. Pereira RM, Tresoldi AT, Hessel G. Isoniazid-induced hepatic failure. Report of a case. Arq Gastroenterol. 2000;37:72-75.

74. Moraes M, González S, García A, Menchaca A. Meningitis tuberculosa en un lactante: Reporte de caso y revisión de la literatura. Archivos de Pediatría del Uruguay. 2016;87:137-142.

75. Kondo S, Miyagawa T. Three cases of tuberculosis children who demonstrated paradoxical worsening during different stage of treatment. Kekkaku : [Tuberculosis]. 2006;81:375-379.

76. Kondo S, Ito M. [Usefulness of cranial and chest imaging in the diagnosis of tuberculous meningitis among infants and young children]. Kekkaku. 2003;78:89-93.
